# ‘See Me, Feel Me’: Prismatic Adaptation of an Alien Limb Ameliorates Spatial Neglect in a Patient Affected by Pathological Embodiment

**DOI:** 10.3389/fpsyg.2018.02726

**Published:** 2019-01-14

**Authors:** Irene Ronga, Francesca Garbarini, Marco Neppi-Modona, Carlotta Fossataro, Maria Pyasik, Valentina Bruno, Pietro Sarasso, Giulia Barra, Marta Frigerio, Virginia Carola Chiotti, Lorenzo Pia

**Affiliations:** ^1^SAMBA (SpAtial, Motor and Bodily Awareness) Research Group, Psychology Department, University of Turin, Turin, Italy; ^2^Manibus Laboratory, Psychology Department, University of Turin, Turin, Italy; ^3^Neuroscience Institute of Turin, University of Turin, Turin, Italy; ^4^San Camillo Hospital, Turin, Italy; ^5^Centro Puzzle, Turin, Italy

**Keywords:** body ownership, bodily self, brain-damaged patients, pathological embodiment, prism adaptation, left neglect

## Abstract

*Pathological embodiment* (E+) is a specific contralesional delusion of body ownership, observed following brain damage, in which patients embody someone else’s arm and its movements within their own body schema whenever the contralesional ‘alien’ arm is presented in a body-congruent position (i.e., 1st person perspective and aligned with the patient’s shoulder). This disorder is often associated with spatial neglect, a neurological syndrome in which patients are unaware of stimuli presented in the contralesional (often the left) space. Capitalizing on previous evidence demonstrating that prismatic adaptation of the ipsilesional arm to right-deviating prisms is effective in ameliorating neglect symptoms, here we investigated whether such amelioration also occurs in E+ patients with neglect when prismatic training is performed by the ‘alien’ embodied arm. Four left neglect patients (one with and three without pathological embodiment) underwent visuomotor prismatic training performed by an ‘alien’ arm. Specifically, while patients were wearing prismatic goggles shifting the visual field rightward, a co-experimenter’s left arm presented in a body-congruent perspective was repeatedly moved toward visual targets by another examiner. In a control condition, the co-experimenter’s arm was moved toward the targets from a body-incongruent position (i.e., 3rd person perspective). Neglect symptoms were assessed before and after training through paper-and-pencil tasks. In the E+ patient, neglect improved significantly more in 1st than in 3rd person perspective training, suggesting that prismatic adaptation of the ‘alien’ embodied arm is effective in modulating spatial representation. Conversely, for control E- patients (not embodying the ‘alien’ arm), we observed more limited improvements following training. These findings indicate that the ‘alien’ embodied arm is so deeply embedded in the patient body and motor schema that adaptation to prismatic lenses can affect multiple processing stages, from low level sensory-motor correspondences, to higher level body, motor and spatial maps, similarly as it occurs in normal subjects and neglect patients without pathological embodiment.

## Introduction

Pathological embodiment is a specific contralesional body ownership disorder, in which brain damaged patients (from now on E+) embody someone else’s arm within their own body schema and are firmly convinced that it is their own arm, whenever it is located in a body-congruent position from a 1st person perspective (Garbarini and Pia, 2013; Pia et al., 2013a, 2016; Garbarini et al., 2013b, 2014, 2015, 2017; Berti et al., 2015; Fossataro et al., 2016, 2018). In a seminal study exploiting the bimanual coupling effect (Franz et al., 1991), Garbarini et al. (2013b) demonstrated that such delusion also extends to the movements of the ‘alien’ embodied arm. When E+ patients are asked to draw straight lines with their right intact hand, while observing the alien embodied hand drawing circles, both shapes tend to show a common distortion (i.e., an ovalization). Importantly, a similar degree of ovalization is observed in healthy subjects when performing the same bimanual task, with their own hands (Garbarini et al., 2013a). These results have been confirmed by further studies (Garbarini et al., 2015; Fossataro et al., 2016) and indicate that E+ patients not only do embody the movements of the alien arm, but also the higher-order consequences of those movements.

In the present study, we aimed at testing whether the embodied arm is so deeply embedded within the patient’s motor system as to modulate also the representation of external space. This is in principle possible because, even though pathological embodiment is double dissociated from several other concomitant deficits, it has often been reported in association with left spatial neglect. Left spatial neglect is a complex neuropsychological syndrome following unilateral brain damage, more frequently to the right than to the left hemisphere, where patients are unaware of stimuli located in the left hemispace, failing to react to and search for them (Halligan et al., 2003). Neglect is typically assessed through a large variety of paper-and-pencil tests: e.g., cancelation tasks (Albert, 1973; Gauthier et al., 1989; Friedman, 1992; Ota et al., 2001; Pia et al., 2013b; Ricci et al., 2016), line bisection tasks (Chatterjee et al., 1992; Milner et al., 1993; Pia et al., 2012), and drawing from memory and copying tasks (Halligan and Marshall, 1993; Rode et al., 2006). Although rehabilitation of neglect is particularly difficult due to the presence of anosognosia, Prismatic Adaptation (hereinafter PA) has been proven to be particularly effective in rehabilitating neglect in the short or medium term (Rossetti et al., 1998; Farnè et al., 2002; Frassinetti et al., 2002; Serino et al., 2007; Striemer and Danckert, 2010; Facchin et al., 2013; Rabuffetti et al., 2013; Ronga et al., 2017a). During classical prismatic training (hereinafter PT), neglect patients perform a series of pointing movements toward visual targets while wearing prisms displacing their gaze rightward. Due to the deviation of the visual field, patients perform some initial rightward pointing errors. However, during the training a compensatory behavior, counteracting prismatic deviation, emerges (i.e., the patient orients his/her pointing movements leftward), allowing patients to reach the visual targets correctly (i.e., PA). In other words, PA consists in a recalibration of visual, proprioceptive and motor inputs, updating inter-sensory correspondences to compensate for the deviated visual information (Redding and Wallace, 2006; Ronga et al., 2018). Interestingly, Ronchi et al. (2011) demonstrated that, in a population of healthy subjects, the simple observation in a first person perspective of repeated pointing errors mimicking those executed during PA induced similar effects to those triggered by classical visuomotor PA, thus indicating that actual execution of pointing movements is not necessary to induce adaptive processes, being sufficient the observation of these same movements. Furthermore, in two recent studies on healthy subjects and neglect patients (Ronga et al., 2017a,b), we showed that an oculomotor PT, only consisting in gaze shifts toward visual targets while wearing prismatic goggles, produces similar, although weaker, after-effects in line bisection to those produced by PA induced through pointing movements (i.e., visuomotor PT).

Capitalizing on the above mentioned results, here we investigated whether and to what extent PA occurs, thus ameliorating neglect symptoms, when the E+ patient is convinced that his/her ‘alien’ embodied arm is moving during PT. In order to answer this question, we administered a visuomotor PT performed by an ‘alien’ arm to one left hemiplegic patient with left neglect and delusional ownership of a left ‘alien’ arm and to a group of three left hemiplegic patients with left neglect but without delusional ownership of the ‘alien’ arm (hereinafter E-). Following PA, we predicted a stronger amelioration of neglect for the E+ patient than for the E- patients. We reasoned that in presence of pathological embodiment, the effects of PA on neglect should be comparable to those observed following visuomotor PT (i.e., stronger), whereas in absence of the delusion, PA effects should be weaker.

## Materials and Methods

### Participants

Five right-brain damaged patients, recruited at the Presidio Sanitario San Camillo, Torino (Italy), were included in the study. Patients details are summarized in Table 1.

**Table 1:** Patients’ information.

					Lesion			Time post					Visual field
Pat.	Sex	Age	Hand	Aet.	(right)	Neuropsychological tests		lesion	E+/E-	HP	HA	P	defects
						Cognitive	BIT						
**1**	F	74	R	I	FTP	MMSE: 22,3	CT: 86/145 BT: 38/81	8 w	**E+**	3	3	Yes	No
**2**	M	65	R	H	FTP	MOCA: 19/30	CT: 87/145 BT: 43/81	12 w	E-	3	3	Yes	No
**3**	M	69	R	I	FTO	MOCA: 13/30	CT: 80/145 BT: 40/81	10 w	E-	3	3	Yes	No
**4**	M	63	R	H	FTP	-	-	2 Y	E-	3	3	Yes	No
**5**	F	74	R	H	FP	MOCA: 10/30	CT: 67/145 BT: 30/81	4 w	**E+**	3	3	Yes	No


*Pat., Patients’ number. Sex: F, female; M, male. Hand indicates hand dominance: R, right; L, left. Aet., etiology: I, ischemia; H, hemorrhage. Lesion (always in the right hemisphere): F, frontal; T, temporal; P, parietal; O, occipital. Neuropsychological tests: MOCA, Montreal Cognitive Assessment; MMSE, Mini Mental State Examination. BIT refers to the Behavioral Inattention Test: CT, conventional tests; BT, behavioral tests. Time post lesion indicates when the experiment was administered after symptom insurgence: w, weeks; Y, years. E+/E-, presence or absence of pathological embodiment. HP, left upper limb hemiplegia. HA, left upper limb hemianesthesia. P, presence or absence of left upper limb proprioceptive deficits.*

Patient 1 (E+), was a 74 years old right-handed (Oldfield, 1971) female who suffered from an ischemic stroke involving right frontal, temporal and parietal lobes. Eight weeks after the stroke, she was initially screened with the Mini Mental State Examination (Measso et al., 1993) in order to evaluate the overall cognitive impairment (range 0–30, patient’s score 22.3). Contralesional motor and tactile deficits were assessed according to a standardized protocol (Bisiach and Faglioni, 1974), whereas proprioception was assessed by means of the joint position matching task in which the patient is asked to recreate a joint angle in the absence of vision (Goble, 2010). The patient displayed upper limb hemiplegia (range 0–3, patient’s score 3), hemianaesthesia (range 0–3, patient’s score 3) as well as a proprioceptive deficit (range 0–1, patient’s score 1). The presence of the pathological embodiment of someone else’s arm was assessed with an *ad hoc* protocol and the patient resulted to manifest the embodiment delusion. Left extrapersonal neglect was assessed with the Behavioral Inattention Test (Wilson et al., 1987) and the Diller cancelation task (Diller and Weinberg, 1977). In both tests, the patient resulted to have left neglect (Diller: left side omissions - right side omissions = 7; Behavioral Tests: 38/81; Conventional Tests 86/145).

Patient 2 (E-) was a 74 right-handed (Oldfield, 1971) male who suffered from a right fronto-parietal hemorrhagic stroke. He was administered the Montreal Cognitive Assessment (Nasreddine et al., 2012) to evaluate the overall cognitive impairment (0–30, score 19) 12 weeks after the stroke. The patient displayed upper limb hemiplegia (range 0–3, patient’s score 3), hemianaesthesia (range 0–3, patient’s score 3) as well as a proprioceptive deficit (0–1, score 1) according to standardized protocols (Bisiach and Faglioni, 1974; Goble, 2010). He did not present pathological embodiment in the administered *ad hoc* protocol but displayed contralesional neglect (Diller: left side omissions - right side omissions = 10; Behavioral Tests: 43/81; Conventional Tests 97/145).

Patient 3 (E-), was a 69 right-handed (Oldfield, 1971) male who suffered from a right fronto-temporal-occipital stroke. He was administered the Montreal Cognitive Assessment (Nasreddine et al., 2012) to evaluate the overall cognitive impairment (0–30, score 13) 10 weeks after the stroke. The patient displayed upper limb hemiplegia (range 0–3, patient’s score 3), hemianaesthesia (range 0–3, patient’s score 3) as well as a proprioceptive deficit (0–1, score 1) according to standardized protocols (Bisiach and Faglioni, 1974; Goble, 2010). He did not present pathological embodiment in the administered *ad hoc* protocol but displayed contralesional neglect (Diller: left side omissions - right side omissions = 13; Behavioral Tests: 40/81; Conventional Tests 80/145).

Patient 4 (E-), was a 63 right-handed (Oldfield, 1971) male who suffered from a right fronto-temporal-parietal hemorrhagic stroke. Two years after the stroke, the patient displayed upper limb hemiplegia (range 0–3, patient’s score 3), hemianaesthesia (range 0–3, patient’s score 3) as well as a proprioceptive deficit (0–1, score 1) according to standardized protocols (Bisiach and Faglioni, 1974; Goble, 2010). He did not present pathological embodiment in the administered *ad hoc* protocol but displayed contralesional neglect (Diller: left side omissions – right side omissions = 15; Behavioral Tests: 40/81; Conventional Tests 80/145).

Patient 5 (E+), included as a control patient for Experiment 2, was a 74 years old right-handed (Oldfield, 1971) female who suffered from a right fronto-parietal hemorrhagic stroke. Four weeks after the stroke, she was administered the Montreal Cognitive Assessment (Nasreddine et al., 2012) to evaluate the overall cognitive impairment (0–30, score 10). The patient displayed upper limb hemiplegia (range 0–3, patient’s score 3), hemianaesthesia (range 0–3, patient’s score 3) as well as a proprioceptive deficit (0–1, score 1) according to standardized protocols (Bisiach and Faglioni, 1974; Goble, 2010). Similarly to Patient 1, she presented pathological embodiment in the administered *ad hoc* protocol and displayed contralesional neglect (Diller: left side omissions – right side omissions = 6; Behavioral Tests: 30/81; Conventional Tests 67/145).

Importantly, all patients did not show any evident visual field defects and had not been exposed to PA before the study. They gave their written informed consent to participate to the study which conformed to the standards required by the Declaration of Helsinki and was approved by the Ethical Committee of the ASL TO 1 of Turin.

### Procedures

The main experiment (Experiment 1) was composed of three sessions, each administered on a different day (no more than 2 days elapsed between two successive sessions). During Session 1, patients were asked to complete four experimental tasks (i.e., *Line bisection, Clock face drawing, Daisy drawing from memory*, and *Copy of daisies*). Before completing once more the experimental tasks, in Session 2 patients performed *3rd Person Perspective Prismatic Training (3rd PT)* and, in Session 3, *1st Person Perspective Prismatic Training (1st PT)*.

Experiment 2 was performed as a control experiment, and aimed at verifying that possible neglect improvements observed in Experiment 1 were specifically due to prismatic adaptation, and not to a general enhancement of attention toward the left hemifield, induced by the prolonged observation of movements performed by the experimenter within the contralesional hemispace. Experiment 2 was composed of three sessions, administered on three consecutive days. During Session 1, the patient was asked to complete three experimental tasks (i.e., *Line bisection, Clock face drawing, Copy of daisies*). In Sessions 2, before completing once more the same experimental tasks, the patient performed a training procedure identical to *1st PT*, except for the fact that the patient did not wear prismatic lenses (*1st PT- sham*, see Training Procedure – Experiment 2 for a detailed description). In Session 3, the patient performed a regular *1st PT*.

#### 1st Person Perspective Prismatic Training (1st PT)

**FIGURE 1:**
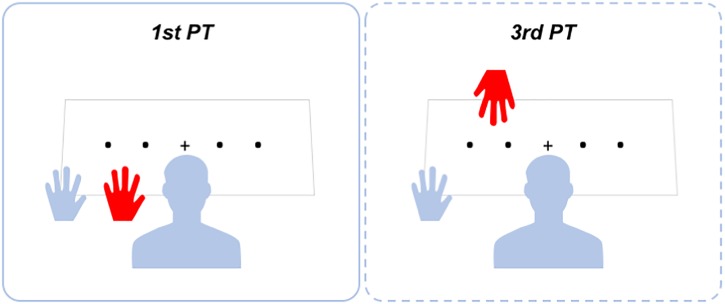
Representation of prismatic training setup.

A representation of training settings is presented in Figure 1. One experimenter (Experimenter 1) stood in front of the patient and gave her/him instructions for completing the PT. The patient sat at a desk, with the contralesional left arm laterally displaced in relation to her/his shoulder. A second experimenter (Experimenter 2) placed her/his own left arm on the desk from behind the patient and aligned it with the patient’s shoulder, in such a way that the experimenter’s arm laid between the patient’s arm and the patient’s body midline. A black cloth covered the patient’s shoulder and both left arms, though leaving the patient’s and the Experimenter 2′s hands clearly visible. In 1st PT setting, Experimenter 2′s hand was perceived by the patient in an egocentric perspective (see Figure 1).

Before starting the training, the patient was asked to report how many left hands she/he could see and to touch with her/his right hand the dorsum of her/his own left hand. During 1st PT, an A3 PT working sheet was placed upon the table. The working sheet had four black dots printed on it, sequentially numbered from 1 to 4 and evenly spaced along the horizontal side of the paper (11.8 cm inter-dots spacing). The working sheet was placed at a distance of approximately 30 cm from the patient’s chest. A black cross (fixation cross) was printed at the center of the paper sheet and was aligned with the patient’s trunk midline. Before starting the training, the patient was asked to close her/his eyes and put on a pair of prismatic goggles equipped with 20-diopter prismatic lenses oriented so as to shift the visual field 11° to the right. The patient was then asked to open her/his eyes to carry out the training. Every 10 s, Experimenter 1 said aloud the number of a dot and passively moved Experimenter 2′s hand toward it. We decided to passively move Experimenter 2′s hand, to reduce the possibility that Patient 1 (E+), affected by pathological embodiment, deceived herself that she regained the ability to actively move her hemiplegic arm during the training. However, at the end of the task, when questioned if she moved her left arm during the training, she answered affirmatively, “…although with the help of the experimenter,” she said.

Pointing movements toward the dots were forty in total (10 per dot). The pointing movements were performed to reflect a real adaptation process. Therefore, the first 10 movements missed the target, showing a rightward pointing error (i.e., in the direction of the prismatic deviation), progressively decreasing from movement 7 to 10. Pointing movements from 11 to 40 correctly hit the target. Pointing errors were initially performed since, in case of simple movement observation, they are known to be crucial for the occurrence of adaptive processes, as demonstrated by Ronchi et al. (2011).

During the task, the patient was instructed to gaze at the moving hand and to focus on the movements. Even though the patient was hemiplegic and fully aware of her/his motor deficit, during PT patients were asked to actively try to move their arm. 1st PT lasted around 7 min.

#### 3rd Person Perspective Prismatic Training (3rd PT)

The patient sat at the desk with his/her own left arm in the same position as for 1st PT. A black cloth covered the arm, leaving the patient’s hand in open view. Experimenter 1 stood in front of the patient. PT was identical to 1st PT except for the fact that it was performed with Experimenter 1′s left hand. In this way, the patient perceived the Experimenter’s arm in an allocentric perspective. Importantly, the patient was always instructed to focus on the moving hand (see Figure 1). 3rd PT had the same duration as 1st PT (i.e., 7 min).

Our predictions were that both 1st PT and 3rd PT would have ameliorated spatial neglect of patients E- in a similar way, due to the effectiveness of ocular movements alone in inducing PA (Ronga et al., 2017a,b). Conversely, in Patient 1 (E+), we expected that 1st PT was more effective than 3rd PT in ameliorating neglect symptoms due to the occurrence -in 1st PT only- of the delusional movements of the embodied ‘alien’ arm. To assess neglect severity, we employed four different tasks, performed before and after PTs.

#### Training Procedure – Experiment 2

In Session 2, during *1st PT-sham*, the patient and the experimenters occupied the same positions, as for *1st PT*, and followed the same procedure described in §1st PT (training duration: 7 min). However, the patient did not wear prismatic goggles. In Session 3, the patient was exposed to a regular 1st PT, as described in §1st PT.

To exclude the occurrence of any adaptive visuomotor process, in Experiment 2, during Session 2 (*1st PT-sham*), no pointing errors were made by the experimenter (Ronchi et al., 2011). Before starting the trainings, the patient was asked to report how many left hands she could see and to touch with her right hand the dorsum of her own left hand. At the end of Sessions 2 and 3, we asked the patient whether she was able to move her left arm: she answered that she could move her own arm thanks to the help of the experimenter.

#### Line Bisection Task

The patient sat at a desk and was instructed to bisect with a pencil ten 18 cm-long horizontal lines printed in the center of an A4 sheet of paper (landscape oriented) and placed at a distance of approximately 30 cm from the patient’s chest. The sheet of paper was aligned with the patients’ body midline and scotched to the desk. The 10 lines to bisect were presented sequentially to the patients, i.e., they were shown one at a time. Severity of neglect was measured in terms of the amount of the rightward bisection error.

#### Statistical Analyses on Line Bisection Tasks

With the aim of verifying the presence of spatial neglect, we performed, for each Patient, a one sample *t*-test on results of Session 1 compared against zero. To correct for multiple comparisons (since five different *t*-tests were performed), we applied Bonferroni correction to the significance threshold, which was set at 0.01 (0.05/5).

In Experiment 1, in order to examine patients’ line bisection performances across sessions, we calculated, for Sessions 2 and 3, the deviation difference from the baseline (Session 1), thus obtaining two measurements (i.e., baseline deviation minus 3rd PT deviation; baseline deviation minus 1st PT deviation) for each patient. Importantly greater deviations from the baseline indicate greater neglect improvements. To directly compare deviation differences following 1st and 3rd PTs between the E+ patient and the group of E- patients, we calculated the ratio between ‘baseline minus 1st PT’ and ‘baseline minus 3rd PT’ deviations. We therefore averaged the scores obtained by E- patients, to obtain a group measurement. Crucially, ratios close to one would indicate that 1st PT and 3rd PT similarly affected patients’ neglect symptoms. Conversely, ratios greater than one would indicate that 1st PT was more effective than 3rd PT in reducing neglect symptoms; ratios smaller than one would indicate the opposite. To investigate possible significant differences between the ratios obtained by E+ patient and the group of E- patients, we performed a Crawford test (two-tailed), specifically designed to test small clinical samples and allowing to compare the performance of a single case against small control groups (Crawford et al., 2010).

In Experiment 2, in order verify whether 1st PT-sham affected neglect symptoms similarly to real 1st PT, we compared Patient 5 line-bisection results obtained in Sessions 2 and 3 to E- control group performances following 1st and 3rd PTs. As a first step, for both Patient 5 and the E- control group, we calculated the deviation difference from the baseline for each session. Furthermore, for Patient 5, we computed the ratio between ‘baseline minus 1st PT-real’ and ‘baseline minus 1st PT-sham’ deviations, whereas, for the E- control group, we employed the ratio between ‘baseline minus 1st PT’ and ‘baseline minus 3rd PT’ deviations. Crucially, if 1st PT-sham had similar effects to actual 1st PT, Patient 5 ratio should be close to 1, and not significantly different from E- ratio, where prismatic adaptation is always employed. Conversely, if 1st PT-sham was not able to reduce neglect symptoms, Patient 5 ratio should be different from 1 and significantly different from E- ratio. To directly test these hypotheses, we performed the same Crawford test (two-tailed) employed in Experiment 1.

Furthermore, we verified whether, following (actual) 1st PT, Patient 5′s performance was comparable to Patient 1′s line bisection results. To do so, we performed a Crawford test (two-tailed), allowing to compare the results of two single cases against each other (Crawford et al., 2010).

#### Clock Face Drawing Task

The patient sat at a desk and had to fill in with numbers an empty circular clock frame (Figure 1; Di Pellegrino, 1995). Omission of one or more numbers occupying the left portion of the clock frame, as well as errors in number disposition, demonstrates the presence of representational neglect (Halligan et al., 1992).

#### Daisy Drawing From Memory Task

The patient was asked to draw a daisy from memory at the center of a blank sheet of paper.

#### Copy of Daisies Task

**FIGURE 2:**
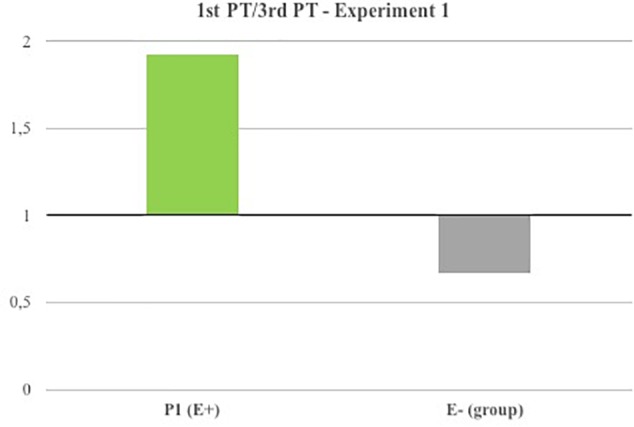
Line bisection results (Experiment 1).

The patient was asked to copy the picture of a flowerpot, composed of two branches of daisies (see Figure 2). Omission of the left flower as a whole or of the left part of the right flower (in presence of a correct copy of the right part of the left flower), are indicative of the presence of egocentric (i.e., referenced to egocentric, body-related coordinates) or allocentric (i.e., referenced to external environment or object-centered coordinates) neglect, respectively (Hillis et al., 1998; Kerkhoff, 2001).

## Results

### 1st and 3rd Person Perspective PT and Pathological Embodiment

Before starting both PTs in Experiment 1 and the training performed in Experiment 2, the Patients were asked to touch with her/his right hand the dorsum of her/his own left hand.

In Experiment 1, during *1st person perspective PT*, Patient 1 (E+) systematically touched Experimenter 2′s arm, which was placed in a body-congruent position (*egocentric perspective*), instead of her own, thus showing embodiment of the experimenter’s arm (Pia et al., 2013a; Garbarini et al., 2014, 2015; Fossataro et al., 2016). Furthermore, at the end of *1st person perspective PT*, Patient 1 (E+) reported that during the training she was able to move her arm thanks to the help of the experimenter. Conversely, all E- patients never touched the experimenter arm, thus indicating that, even when Experimenter 2′s arm was placed in an egocentric perspective, they never embodied it and they were always able to correctly discriminate between their own and the experimenter’s arm. At the end of *1st person perspective PT*, E- patients reported to see a stranger’s arm moving alongside their own during the training.

In 3rd person perspective PT, where the experimenter’s arm was not placed in a body-congruent position, all patients were always able to successfully discriminate between their own and the experimenter’s arm.

In Experiment 2, Patient 5 (E+) always touched Experimenter 2′s arm, which was always placed in the same position as for *1st person perspective PT*, instead of her own. This observation demonstrates that in Sessions 2 and 3 the patient embodied the experimenter’s arm (Pia et al., 2013a; Garbarini et al., 2014, 2015; Fossataro et al., 2016). At the end of both tasks (1st PT and 1st PT-sham), Patient 5 reported that during the trainings she was able to move her arm thanks to the help of the experimenter.

### Experiment 1

#### Line Bisection Task

In Session 1, all patients showed a significant rightward bisection error, indicating the presence of spatial neglect (average rightward deviation as compared to midline ± standard deviation in cm; one sample *t*-test compared against zero: patient 1, 2.65 ± 0.52 cm, *t* = 15.95, *p* < 0.001; patient 2, 1.20 ± 0.36 cm, *t* = 10.66, *p* < 0.001; patient 3, 0.83 ± 0.57 cm, *t* = 4.07, *p* = 0.001; patient 4, 0.7 ± 0.61 cm, *t* = 3.58, *p* = 0.006; patient 5, 0.92 ± 0.48 cm, *t* = 6.12, *p* < 0.001).

Deviations from the baseline recorded following 1st PT and 3rd PT, as well as 1st PT/3rd PT deviations (ratios) are reported in Table 2. Overall, both PTs were able to reduce neglect symptoms in all patients. However, as shown in Figure 2, Patient 1 (E+)’s ratio was greater than 1 (1.93), thus indicating that 1st PT was more effective in reducing the line-bisection error as compared to 3rd PT. Conversely, in the E- control group, the calculated ratio was close to 1 (average ± standard deviation: 0.68 ± 0.25), suggesting that 1st and 3rd PTs similarly affected patients’ performances. Crucially, the Crawford test indicated that Patient 1 (E+) ratio was significantly different from E- control group’s ratio (*t* = 4.33, *p* = 0.049; effect size Z_cc_ [plus 95% CI] = 5.00 [0.581 to 9.713]).

**Table 2:** Patients’ line-bisection scores.

P	1st PT	3rd PT	1st PT/3rd PT
**1 (E+)**	1.29	0.67	1.92
**Av. E-**	0.89 ± 0.76	1.23 ± 0.7	0.68 ± 0.3


*Deviations from the baseline recordings following 1st PT and 3rd PT, as well as 1st PT/3rd PT deviations (ratios) are reported. 1, patient’s number; Av. E-, average of E- patients’ scores; 1st PT, scores obtained following 1st PT; 3rd PT, scores obtained following 3rd PT; 1st PT/3rd PT, ratios between deviations following 1st and 3rd Pt.*

#### Clock Face Drawing and Copy of Daisies Tasks

Drawings were shown to three judges unaware of the experimental condition in which the drawings were collected (Ronga et al., 2017a). Judges were asked to evaluate each drawing by assigning a neglect score ranging from 0 to 3 attributed to the left and right side of each copied object (3 = severe neglect; 2 = moderate neglect; 1 = mild neglect; 0 = absence of neglect) (Pia et al., 2004). Judges’ drawing scores are reported in Table 3 (Judges’ agreement was always between 60 and 70%).

**Table 3:** Patients’ drawings scores.

	Clock drawing						Daisy from memory						Copy of daisies					
	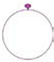						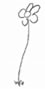						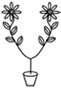					
			
	Left			Right			Left			Right			Left			Right		
	S1	1st	3rd	S1	1st	3rd	S1	1st	3rd	S1	1st	3rd	S1	1st	3rd	S1	1st	3rd
**1 (E+)**	**3**	**0.7**	**1.7**	**1.7**	**1.7**	**1.7**	**2.3**	**0.3**	**3**	**1.3**	**1**	**3**	**3**	**0.3**	**2.7**	**0.7**	**0.3**	**0.3**
**2**	3	1.3	1.3	1.3	1.7	1	2.7	3	2.7	1	1	0.3	3	2.7	2	2	1.3	1.7
**3**	1	0.3	0.3	1	0.3	0.3	0.7	0.7	0.3	1	1	0	3	3	1	1	1	0.7
**4**	2	0	1.7	0	1	1.3	0.7	1.3	0.7	0	0	0.7	2	1.3	0.3	0.7	0	0.3
**Av. E-**	**2**	**0.5**	**1.1**	**0.7**	**1**	**0.9**	**1.4**	**1.7**	**1.2**	**0.7**	**0.7**	**0.3**	**2.7**	**2.3**	**1.1**	**1.2**	**0.8**	**0.9**


*For each patient, the scores assigned by the judges were averaged for each side of the drawings (left/right) and for each session (Session 1/post 1st PT/post 3rd PT). 1–4, patients’ numbers; Av. E-, average of E- patients’ scores; Left, drawings’ left side; Right, drawings’ right side; S1, scores obtained in Session 1; 1st, scores obtained following 1st PT; 3rd, scores obtained following 3rd PT.*

**FIGURE 3:**
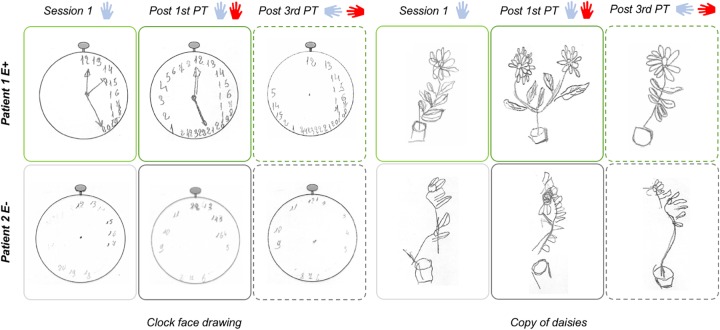
Clock face drawing task and copy of daisies results (Experiment 1).

Spatial neglect is evident in the majority of the drawings collected in Session 1. Importantly, Patient 1 (E+) improvements appeared larger following 1st PT than 3rd PT. Conversely, E- control group patients had similar performances following both PTs. A representative sample of the patients’ drawings is represented in Figure 3.

It is interesting to note that Patients 1 and 2, at least in Session 1, appeared to disregard small numbers in the clock face drawing (see Figure 3, left panel), even though they are represented in the right portion of the clock. As pointed already pointed out (Aiello et al., 2012), this impairment might be due to a pivotal role of right hemisphere in representing small numerical magnitudes.

### Experiment 2

#### Line Bisection Task

**FIGURE 4:**
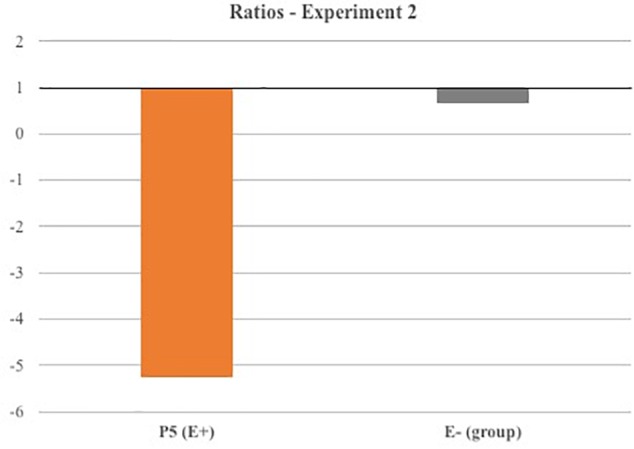
Line bisection results (Experiment 2).

In Session 1, Patient 5 showed a significant rightward bisection error, typical of spatial neglect (average rightward deviation as compared to midline ± standard deviation in cm; one sample *t*-test compared against zero: patient 5, 0.92 ± 0.48 cm, *t* = 6.12, *p* < 0.001). Following 1st PT-sham, the patient did not show any bisection improvement, as the rightward bias was on average slightly higher (1.04 ± 0.41 cm). Following (actual) 1st PT, the patient showed a clear line-bisection bias improvement (0.29 ± 0.30 cm). Therefore, the calculated ratio between 1st PT/1st PT-sham deviations was much smaller than 1 (-5.25), thus suggesting a different modulation of Patient 5′s performances following 1st PT-sham and (actual) 1st PT (see Figure 4). The Crawford test revealed that Patient 5′s ratio was significantly different from E- control group ratio, where 1st PT and 3rd PT similarly affected the results (*t* = 19.75, *p* = 0.002; effect size Z_cc_ [plus 95% CI] = -22.808 [-43.830 to -3.585]). This result seems to indicate that (actual) 1st PT induced significantly greater effects on Patient 5 performances as compared to 1st PT-sham (which in contrast did not affect patient’s line bisections). Importantly, another Crawford test highlighted that, following (actual) 1st PT, Patient 5′s performance was not significantly different from Patient 1 (E+)’s results (*t* = 0.57, *p* = 0.627; effect size Z_cc_ [plus 95% CI] = 0.569 [0.091 to 1.094]), thus suggesting that both E+ patients showed similar improvements following (actual) 1st PT.

#### Clock Face Drawing and Copy of Daisies Tasks

Judges’ drawing scores are reported below (Judges’ agreement was between 70 and 100%).

Clock face drawing: Session 1, left side: 2.3; right side: 1. Post 1st-sham, left side: 1.3; right side: 1. Post 1st PT: left side: 0.3; right side: 0.

Copy of daisies: Session 1, left side: 1; right side: 0.67. Post training, left side: 1.67; right side: 0.33. Post 1st PT: left side: 0.3; right side: 0.

Overall, the results of Experiment 2 confirmed the finding of Experiment 1, showing a reduction of neglect symptoms for E+ patients following 1st PT. Importantly, no significant improvements were observed following 1st PT-sham (except for a small reduction of representational neglect limited to the clock face drawing). Actually, performance in the copy of daises and in line-bisection tasks slightly worsened following 1st PT-sham.

Altogether the present results seem to indicate that neglect improvements observed in Experiment 1 cannot be ascribed to a general enhancement of attention in the contralesional space related to mere observation of the movements made by the experimenter, but are the consequence of prismatic adaptation.

## Discussion

In the present study, we investigated whether visuomotor prismatic training with an ‘alien’ embodied arm is effective in ameliorating neglect symptoms similarly to what is observed when the own real arm moves (Rossetti et al., 1998). In order to answer this question, we compared the performance in a number of paper-and-pencil tasks pre- and post-training in a patient with (E+) and in a group of patients without (E-) pathological embodiment of an ‘alien’ arm (Experiment 1). Our results showed that PT with the ‘alien’ arm ameliorated neglect symptoms more in the E+ patient than in control E- patients, but only when PT occurred with the alien arm in a body-congruent position. In particular, in the line-bisection task, while control E- patients showed a similar neglect improvement following both 1st PT and 3rd PT, the E+ patient displayed a significantly different pattern, showing a greater reduction of neglect following 1st than 3rd PT. Similarly, E+ patient’s left sided omissions in the drawing tasks decreased more following 1st PT than 3rd PT, whereas control E- patients displayed similar performances after both types of PTs (as evidenced by the drawings’ qualitative analyses). Altogether these results suggest that, for the E+ patient, 1st PT seems to be as effective as traditional visuomotor PT, whereas in absence of a body ownership delusion, the effects of PT are weaker and may be related to the influence of a purely oculomotor prismatic adaptation mechanism, as demonstrated by Ronga et al. (2017a). Furthermore, when PT was performed without prisms in a control E+ patient, no relevant signs of neglect amelioration were apparent (Experiment 2), thus indicating that the effects observed in Experiment 1 are specifically related to prismatic adaptation processes and cannot be ascribed to a non-specific enhancement of attention induced by the simple observation of limb movements in the contralesional space.

A first key point to address is the stronger amelioration of neglect following 1st than 3rd PT in our E+ patient. Indeed this result is not trivial but, rather, consistent with previous data showing that the delusional body ownership occurs only under certain circumstances. More specifically, it has been repeatedly demonstrated that the pathological embodiment of the ‘alien’ arm occurs only if some constraints in terms of postural and body-like appearance are satisfied. With respect to posture, the delusion manifests only if the ‘alien’ arm is in a shoulder-compatible position and internal to the patients’ left arm, but not if it is either external to the patients’ arm or in a 3rd person perspective (Berti et al., 2015; Pia et al., 2016; Garbarini et al., 2017). Crucially for the present study, whenever the above-mentioned postural constraints are satisfied, patients embed not only the ‘alien’ arm but also its active and passive movements (Pia et al., 2016). Hence, these data are consistent with the different effects of 1st and 3rd person perspective ‘alien’ arm PT described in the present experiment.

A further important point to discuss regards the nature of the effects triggered by PT on the ‘alien’ embodied arm. As mentioned above, it has been demonstrated that the ‘alien’ arm is embodied not only in the patients’ somatosensory system but also in their motor system. A previous study (Garbarini et al., 2013b), showed that in E+ patients, the movements of the ‘alien’ embodied arm interfere with the actual movements of the ‘healthy’ arm, similarly as it occurs in healthy participants (Franz, 2003; Swinnen et al., 2003; Garbarini et al., 2013a; Piedimonte et al., 2014). Moreover, subsequent studies examined whether the effects of the ‘alien’ arm movements extend to the patient’s personal and peripersonal space (Garbarini et al., 2015; Fossataro et al., 2016). A first study (Fossataro et al., 2016), asked whether these movements affect E+ patients’ peripersonal space and demonstrated that the hand-blink reflex [i.e., the reflex triggered by the electrical stimulation of the median nerve which, in healthy participants, increases when the hand is close to the face (Sambo et al., 2012)] occurred even when the ‘alien’ embodied hand was moved close to the patient’s face. Another study, investigated if the ‘alien’ hand movements can modulate E+ patients’ personal space representation (Garbarini et al., 2015). Capitalizing on the evidence that active tool use can reshape the body metric in normal subjects (Sposito et al., 2012), they showed that tool use training performed with the ‘alien’ embodied arm has effects on the body metric of E+ patients comparable to those described for normal subjects. In the present study, we went a step further by showing that the movements of the ‘alien’ embodied arm are so deeply embedded within the patient’s motor system as to affect not only the representation of the body but also the representation of the external space. In other words, when performed with an ‘alien’ embodied hand, PT can induce a recalibration of visual, proprioceptive and motor inputs influencing higher level spatial representation, similarly to what is observed after traditional visuomotor PA.

In conclusion, our results show that pathological embodiment is not limited to the body and motor schema but extends to the sensorimotor consequences of PA upon higher level spatial representation, suggesting that pathological embodiment similarly affects multiple cognitive levels, from low level sensory-motor correspondences, to higher level body, motor and spatial maps.

## Author Contributions

IR, LP, FG, and MN-M designed the study. Data were collected by VB, CF, IR, PS, MP, and VC. All the authors contributed to data interpretation and manuscript preparation.

## Conflict of Interest Statement

The authors declare that the research was conducted in the absence of any commercial or financial relationships that could be construed as a potential conflict of interest.
